# Hearing impairment and nightmares: a theoretical insight

**DOI:** 10.1186/s40064-015-1579-1

**Published:** 2015-12-18

**Authors:** Maria Francisca Rego, Ivone Duarte, Rui Nunes

**Affiliations:** Department of Social Sciences and Health, Faculty of Medicine, University of Porto, Alameda Prof. Hernâni Monteiro, 4200-319 Porto, Portugal

**Keywords:** Deafness, Nightmares, Trauma, Dissociation

## Abstract

The aim of this article is to address the issue of nightmares in the deaf population, given that there are no documented studies on this matter to the best of our knowledge. The study of nightmares in the deaf population is of high relevance given their specific characteristics (impossibility of verbalisation) and the lack of studies with this population. Nightmares are dreams of negative content that trigger an awakening associated with a rapid return to a full state of alert and a persistent feeling of anxiety and fear, which may cause significant distress. Various studies show that the deaf population has dreams with more negative imagery and emotions, are more exposed to interpersonal traumas and have higher rates of dissociation, than hearing people. These concepts seem to be connected given that, in the presence of traumatic events, dissociation may act as a defence mechanism and nightmares may operate as an adaptive coping strategy.

## Background

Dreams have the function of making connections, guided by emotion, between traumatic events and life memories. It is suggested that dreaming has a quasi-therapeutic function, as it allows the making of connections in a safe place—rapid eye movement (REM) sleep and consequently noting similarities between life’s different domains, possessing an integrative role that is most easily observed after trauma (Hartmann 1998).

In general, nightmares tend to follow trauma experiences and are a common issue among the population in general (Krakow et al. 2004; Langston 2007), although their prevalence tends to decrease with age, being more frequent during childhood (Abdel-Kalek 2010). Nightmares have an adaptive role since they tend to disappear as the trauma is gradually resolved (Hartmann 1998). When an individual faces a traumatic experience, nightmares tend to emerge, portraying not the actual detailed event but the emotions experienced by the dreamer, so that the experience can be integrated into life’s narratives (Hartmann 1998; Rego 2014). However, the presence of recurrent nightmares means that the dreamer is not being able to make any new connections between the traumatic and the old memories (Hartmann 1998), given that traumatisation leaves the subject with limited symbolism of the experience. Therefore, the trauma still exists as a presence that threatens to intrude onto the conscious mind and bring the subject back to a resembling situation of the traumatic event (Varvin et al. 2012).

Several studies have documented higher rates of interpersonal traumatic experiences among the deaf population, compared to the hearing population. However, no documented studies have explored nightmares in this population, and given its high relevance at a clinical and research level, this review focuses on the issue of nightmares in the deaf population.

## Dreams: a conceptual approach

Among the various theories on dreaming effect, many have suggested dreams as enablers to obtain insight into one’s emotional and interpersonal life (Blechner  2001; Edwards et al. 2013; Freud 1953). They are considered to be picture metaphors that allow the dreamer to make connections and note similarities between different life domains (Hartmann 1998). Thus, the material in our dreams is always personally relevant; even when a scene never actually happened, there is always a disguised underlying meaning (Freud 1953; Hartmann 1998).

Among the various theories, Hall and Nordby (1972) formulated the continuity hypothesis, where dreams are viewed as continuous with waking life. In other words, the dream and waking world are one, and the person remains the same as do his/her personality and basic beliefs; however it is his/her wishes and fears that determine the actions and thoughts in everyday life, as well as the dreams’ content. Even so, these authors differentiate the overt behaviour (acting out) and covert behaviour (thoughts, feelings, fantasies) present in dreams, which explains why sometimes the dreamer is able to do things in the dream she/he has never done before.

Hobson and Schredl (2011), on the other hand, refer to the discontinuity of dreams, according to which dreams include experiences that were never experienced by the dreamer in waking life, or include dream elements (persons, places) that are different compared to waking life. Nevertheless, these aspects are related to creativity in dreaming, which is not thematically “continuous” with waking life, but is on the level of emotions, for example scene-shifts, condensations, bizarreness, daydreaming, reverie and fantasy (Hartmann 2011a; Schredl 2012). These contents might be “cognitive glitches”, maybe related to the relative deactivation of the prefrontal cortex during REM sleep. Here, the brain is hyper-associative and not capable of congruent schemes, creating scene shifts, or known settings with new elements and so on (Hartmann 2011b; Hobson et al. 2000).

As for dreaming of experiences that were never experienced in waking life by the dreamer, some researchers have attempted to understand this phenomenon in specific populations (ex: mute-deafs communicating; congenital paraplegics walking or congenital blinds seeing). For instance, a study on congenitally blind subjects found the existence of visual imagery in dreams, independent of visual perception, suggesting a possible dissociation of frontal and parietal areas in dreams, as congenitally blind subjects were not able to describe verbally the visual content of their dreams, but were able to provide a graphical representation of the content through drawing (Bertolo et al. 2003). According to these results, the congenitally blind, who had never experienced sight, were able to visualize. The authors hypothesized that blind subjects can produce virtual images, as their dreams correspond to the activation of visual cortical regions. Experience is considered essential for both visual imagery and for visualisation, and visualisation without previous experience would indicate the existence of visual imagery independent of visual perception, implying that born-blind subjects are capable of using other sensory modalities to integrate these inputs via visual system and produce concepts capable of graphical representation. However, it is not known how the reported images in dreams were constructed in the brain and to what extent these images were based on true visual imagery and not on learned behavioural reports (verbalism) (Bertolo et al. 2003).

Another study with congenitally blind subjects found that they experienced significantly more nightmares than the other groups (late blind and sighted control subjects) (Meaidi et al. 2014). Recent theories regarding the function of dreaming point to a more evolutionary purpose of dreaming and nightmares. They state nightmares can be seen as a threat simulation, in other words, as a way for the human mind to adapt to the threats of life, giving an opportunity for the individual to rehearse the threat perception and cope with it (Revonsuo 2000; Valli et al. 2005). The reports of nightmare content of the congenitally blind subjects showed that the themes present were threats that they often dealt with in their waking lives (Meaidi et al. 2014). However, it is also suggested that an increased nightmare frequency in these individuals may also reflect experiences from an early life, internal organisation and differences in coping and modulation of intense feelings. The results from this study are in accordance with the hypothesis that dreaming and waking life are closely associated, however this association should not be overrated, as dreams have multiple layers of meaning that are not only determined by external conditions but also by internal life and past experiences (Meaidi et al. 2014; Hartmann 1998).

Focusing on other populations, Voss et al. (2011) study about dream reports of congenitally paraplegic and deaf-mute individuals suggested that both models of continuity and discontinuity are simplified approaches of dream analysis, as sometimes elements of waking life that appear in the dream world are out of context and dysfunctional. Descriptions of the sensorial experiences of both the congenitally deaf-mute and congenitally paraplegic were found, and they could not be distinguished from those of non-handicapped subjects. Deaf-mute subjects described themselves as talking and experiencing hearing in terms that were indistinguishable from the hearing control group and a phenomenological experience of movement in the paraplegic individuals was described, in which no differences were found in its frequency compared to those of the non-handicapped controls. It is suggested that our sensorial perceptions in dreaming are not subjected to constant adjustment with external world parameters, tending to be a broader and less restrained experience than waking life. Dreaming allows us to experience speech without actually speaking and without the need of reassurance through hearing that our message was received, or experience movement without visual feedback. Thus, sleep provides an opportunity for sensorial exploration by our imagination that is not limited by our actual physical performance capacity. However, virtual movement and speech in dreams does not require to be physically carried out. For instance, it was explained by a deaf-mute individual that speech in dreaming can be experienced without sign language and without speech, as if it were telepathy, which makes sense given that she/he never really experienced speech or hearing in waking life (Voss et al. 2011).

Hobson and Schredl (2011) try to explain these phenomena suggesting that dreaming is a preplay from early on (birth or before birth), speculating that these topics are hard-wired and encoded experiences. In addition, (Schredl 2012) hypothesised that while a person is watching or witnessing real life situations, TV, video games, for example, information processing done by mirror neurons in the waking state might reflect on the dreams’ “discontinuous” elements (Gackenbach et al. 2011; Schredl 2012; Van den Bulck 2004). In general, we can point to the fact that there are no discontinuities at the level of emotions, given that the dreamer is one person with one set of emotions that influence mental processing, which is most evident in dreaming (Hartmann 2011a). Cartwright et al. (2001) have suggested that dream patterns occur which depend on waking personalities and on defence mechanisms, supporting the individual in the ability to form dreams that connect present emotions related to disturbed experiences to other images.

## Deafness, traumas and nightmares: a psychological approach

Deafness is currently considered a public health problem as it interferes permanently with a person’s development. The causes of deafness may be genetic, environmental or a combination of both. At least 50 % of all childhood deafness can be attributed to some genetic factor (Feinmesser et al. 1986) and approximately 50 % is attributed to environmental factors (Graham 2004).

There are two models related to deafness, a medical and a cultural one (Nunes 2006). The medical perspective distinguishes congenital deafness from adventitious deafness, which refers to hearing loss acquired at birth or later in life (Braden 1994; Turkington and Sussman 2004). From a psychological perspective, this distinction is made between prelingual and postlingual deafness (Braden 1994). The prelingual stage includes the time period prior to the acquisition of spoken language, although this period is not well defined due to differences in normal language development among children (Braden 1994). These individuals are often unable to develop intelligible speech (Schein 1989). People who become deaf after the development of normal speech are considered postlingually deaf and a distinction can be made between childhood deafness (age onset before adolescence), prevocational deafness (onset before nineteen), adult onset and elderly onset (Schein 1989). Postlingual deafness is often experienced as psychologically traumatic and may result in various interpersonal and work-related problems (Raifman and Vernon 1996). The medical model tends to view deafness as a disability or impairment that needs to be fixed (Lane et al. 1996) and so various devices have been created, including hearing aids, cochlear implants, speech therapy and oral education, in order to facilitate the integration of deaf people in our society (Austen and Coleman 2004).

The cultural view considers deaf people to be a cultural and linguistic minority Most deaf people who embrace their deafness are prelingually deaf and belong to the deaf community (Deaf world), which provides them a sense of belonging and social support (Austen and Coleman 2004). Culturally deaf people reject the term “hearing impairment” because it implies that there is something wrong that needs to be fixed, and in their opinion being deaf should only suggest a person’s inability to hear (Schein 1989).

Deaf children may be raised in different family environments: deaf families or hearing families. In deaf families, deaf children grow up in an environment that promotes optimal social, emotional, psychological, linguistic and cognitive development (Marschark 1993). However, over 95 % of deaf children are born in hearing families (Mitchell and Karchmer 2004). Less than 10 % of hearing parents with deaf children learn sign language and these communication barriers unable parents to provide supportive parenting and accurate feedback regarding social appropriate environments (Gulati 2003; Marschark 1993; Schild 2007). This results in deaf children growing up in communication-inaccessible and language-deprived environments (Gulati 2003; Marschark 1993), resulting in social exclusion from family interactions and therefore promoting social isolation (Gulati 2003; Schild 2007).

Poor social conditions, such as negative parenting behaviour, family dysfunction and psychopathology, parental poverty, early separation from parents and low education levels (Shalev 1996) are related to the vulnerability of becoming traumatised, factors which are extremely prevalent among deaf individuals (Marschark 1993; Padden and Humphries 2005). The lack of social support and information about a traumatic event increases factors, as suddenness, unpredictability, uncontrollability, incongruence, and novelty of the experience, which have been found to increase traumatisation (Ozer et al. 2003). Prior knowledge about the trauma has a desensitisation effect and is an important part of the recovery process (Kalayjian et al. 2002). Information deprivation trauma appears specially to the deaf population because usually deaf children grow up in hearing environments and are communication-inaccessible and language deprived (Gulati 2003; Marschark 1993), given that only a few percentage of family members learn sign language (Gulati 2003; Marschark 1993) and most deaf people have low reading skills which limits the access to written information (Wolk and Allen 1984). Prelingual deafness, in general, may increase a person’s isolation, language deprivation, lack of learning opportunities, etc., which may influence how a person copes with life stressors (Marschark 1993; Schild 2007).

Treatment methods have been developed to overcome deafness, namely hearing aids and cochlear implants. Hearing aids amplify and transform sounds so they may be detected by damaged ears. Cochlear implants are considered one of the most important technological advances in the rehabilitation of deafness (Eisenberg et al. 2006). They are surgically implanted electronic devices that enable individuals with severe to profound hearing loss to regain or access some hearing. It bypasses damaged portions of the inner ear and stimulates the auditory nerve, allowing the perception of sound and helping deaf people understand speech, though hearing through a cochlear implant is different from normal hearing and it takes time to learn/relearn. Nevertheless, a great variability remains between subject outcomes, as some cochlear implant recipients may obtain only a greater awareness of environmental sounds and others may achieve speech recognition, telephone use and can integrate easily into the hearing world (Zaidman-Zait 2010). Several factors may influence the final outcome of the implant, such as the aetiology of deafness, the age at which deafness is identified, age at the time of the implant, the presence of residual hearing, the process of the auditory rehabilitation and family participation in the therapeutic process (Duarte et al. 2014; Zaidman-Zait 2010).

Cochlear implants can improve physiological communication in the most severe deaf cases, leading to educational, social and psychological benefits, such as higher self esteem, regulation of attention and behaviours, improved parent–child relationships and quality of life, socializing with peers, richer context of communicative experiences, and so on (Duarte et al. 2014; Stein 2007; Weisel and Kamara 2005; Zaidman-Zait 2010). Also, a recent study that carried out a sleep analysis with intra-cochlear implant OFF–ON found shifts in sleep organisation and, in addition, subjects reported that the night they slept with the implant ON was more restorative, probably due to the significant increase in the stage four sleep (Velluti et al. 2010).

Duarte et al. (2014) study found higher levels of quality of life in hearing children, followed by deaf children with cochlear implant with a very small difference, and finally deaf children with significant differences. However, deaf children, both with or without an implant, described feeling discriminated against by their peers, which could be characterized as bullying. The lack of effective communication is the greatest barrier to the adaptation of the deaf person. Usually deaf children receive little explanation about social and emotional behaviours, which leads to difficulties in self-regulation and in understanding social interactions, and this lack of opportunities for participating in a social environment and interacting with peers is a major determinant of a child’s social and emotional development.

Deaf people who lack a solid language foundation often display varying degrees of verbal and cognitive deficits, even with a normal intelligence quotient (IQ) performance, such as feelings of confusion, obsession and being overwhelmed; lack of insight into psychological processes; lack of knowledge about social rules and emotional and social deficits, which may decrease quality of life and the inability to answer basic questions about their lives (Gulati 2003). These deficits and difficulties may explain, among other factors, the relationship between language skills and somatoform dissociation (Freyd 1994; Schild and Dalenberg 2012).

Dissociation is frequently associated with trauma-related avoidance symptoms (Cardeña and Weiner 2004). Deaf people in general, regardless of the trauma exposure and level of traumatisation, are more likely to dissociate than hearing people (Schild 2007). It is possible that early language deprivation and social isolation may have increased a deaf person’s overall vulnerability for dissociation (Freyd 1994). Furthermore, insecure attachment relationships, emotional neglect, neurobiological disturbance, substance abuse and panic attacks have been identified as possible risk factors for dissociation (Briere 2006), aspects which may be more prevalent in the deaf population (Marschark and Clark 1998; Schild and Dalenberg 2012). It is necessary to share traumatic experiences in order to organize them more consciously. However, in deaf subjects the traumatic information cannot be shared, either due to the lack of social confidants or access to sign language therapist, increasing the probabilities of dissociation (Freyd 1994).

Dissociation as a defence may be more present in the deaf individual than in a hearing one. Dissociation allows the disconnection between the internal and external environment, thus the external environment may be perceived in a distorted manner. In a way, it is easier for deaf people to disconnect from their external environment by use of the body to consciously avoid receiving external information, i.e. closing their eyes, which may be an alternative to or an antecedent of dissociation (Stern 1997; Schild and Dalenberg 2012). Furthermore, the presence of post traumatic stress disorder is associated with psychoform and somatoform dissociation in deaf people (Schild and Dalenberg 2012).

It can be stated that deaf people are exposed to interpersonal traumas at a much higher rate than hearing people (Vernon and Miller 2002). Several research studies found that sexual abuse and maltreatment among deaf children and people with disabilities is more prevalent (50 %) than among non-disabled hearing people (10–25 %) (Kvam 2004; Schild 2007; Vernon and Miller 2002). Moreover, since deaf people often have additional neurological and developmental disorders, this may aggravate symptoms that develop following exposure to traumatic events (Diaz et al. 2013).

Post traumatic stress disorder in deaf people may be better conceptualized as a dimensional construct (Solomon et al. 1996), as the validity and reliability of the diagnosis in this population is highly questionable due to the extreme heterogeneity of deaf individuals, absence of valid measures, lack of research in this population and few qualified mental health professionals who provide service to the deaf (Black and Glickman 2006; Schild 2007). For instance, it is important to consider that exaggerated startle responses, which are most commonly triggered by noise, are usually not present in deaf people. Instead, they may simply avoid situations that could provide a visual reminder of the trauma (Schild 2007). Other triggers such as tactile stimulation, temperature, conditioned odours and pain may also be easily avoided than auditory cues (Van der Kolk 1996). In general, deaf patients show a different diagnostic profile than hearing patients, and so the use of culturally competent and fluent sign-language clinicians should be considered in order to provide a more accurate diagnosis and a best practice for the care of deaf individuals (Diaz et al. 2013).

Post-traumatic reactions in deaf people manifest in various forms: expression of symptoms of dissociation; re-experience and avoidance after exposure to potentially traumatising events, which consequently result in symptoms of depression, anger, irritability, sexual concerns, tension reduction behaviours, alcohol and drug problems, as well as distinctive trauma symptoms like strange dreams, feeling disconnected from one’s past, feelings of not being understood, minimisation of traumatic events, obsession about revenge, engaging maladaptive behaviours and feeling hopelessness (Schild 2007; Schild and Dalenberg 2012; Van der Kolk and McFarlane 1996). Previous research has acknowledged a strong relationship between childhood trauma and the development of dissociative symptomatology as a defence mechanism, in which nightmares are considered one of its adaptive coping strategies in trauma (Agargun et al. 2003).

One of the most common post-traumatic sleep disturbances are nightmares (Krakow et al. 2004; Langston 2007). They are described as vivid dreams that normally last 4–15 min and end when a person awakes due to the rapid return to full alertness, together with the feelings of fear and anxiety and recalling the dream vividly, which usually leads to sleep disruption or difficulty in returning to sleep (Abdel-Khalek 2010; American Academy of Sleep Medicine 2005; American Psychiatric Association 2013). Fear has often been related to the neurobiological response of hyperarousal, and so it seems reasonable that the fear felt in nightmares would more often result in the awakening of the dreamer (Zadra et al. 2006). Nightmares usually occur in the second half of the REM sleep and rarely involve motor or behavioural activity due to the loss of muscle tone that occurs in this sleep stage (American Psychiatric Association 2013; Langston 2007).

Studies concerning dream content in congenital deafness found that their dream experiences, besides reflecting the way they view and experience their waking lives (Gilliland and Stone 2007), have significantly more negative events, emotions and more aggressive interactions, as well as frequent verbal communication (Pires 2009; Pires, et al. 2007; Voss et al. 2011), suggesting a clinical utility in therapy (Gilliland and Stone 2007).

Despite the similarities between bad dreams and nightmares, they differ in the regulation of affect levels (American Psychiatric Association 2013; Levin and Nielson 2009). Nightmares reflect increased deficits in emotional regulation, contain more negative dream imagery, more references to death, aggression and hostility and are significantly more intense and therefore more likely to be more distressing than bad dreams. Bad dreams, on the other hand, contain more markers of emotional processing, as articulated and modulated expressions of emotions (Fireman et al. 2014).

Frequent nightmares are generally an expression of a disturbance in sleep structure, which may have serious consequences in health. They can be related to somatic or mental symptoms (anxiety, mood disorder, psychotic disorders, adaptation disorders, personality disorders, suicide) and a bad quality of life (life stressors and traumas) (Abdel-Khalek 2010; Langston 2007; Martinez et al. 2005), but may also emerge as an independent psychopathological problem (American Psychiatric Association 2013). Studies have shown higher prevalence of musculo-skeletal and gastrointestinal symptoms in prelingually deaf persons (Nygren et al. 2001), as well as more depressive symptoms and sleep disturbances, as insomnia, in elderly prelingually deaf people (Wergren-Elgström et al. 2003). Also, higher rates of impulsivity (Landsberg and Diaz 2010; Sheppard 2013), anxiety (Diaz et al. 2013; Fellinger et al. 2012), psychoses (Fellinger et al. 2012), depression (Diaz et al. 2013; Fellinger et al. 2012; Sheppard and Badger 2010; Wergren-Elgström et al. 2003), inappropriate social behaviour (Black and Glickman 2009; Landsberger and Diaz 2010), suicide and mutilation (Landsberg and Diaz 2010; Sheppard and Badger 2010; Sheppard 2013), post traumatic stress disorder (Glickman 2009; Sheppard and Badger 2010), mood disorders (Black and Glickman 2009; Glickman 2009; Shapira et al. 1999), developmental disorders (Black and Glickman 2009; Landsberger and Diaz 2010; Glickman 2009) and personality disorders (Glickman 2009; Appleford 2003; Landsberger and Diaz 2010) were found in the deaf population.

The consequence of suffering from nightmares is mostly related to the distress caused to the subject, due to the distressing dreams that provoke awakenings together with feelings of fear and anxiety. This results in fear of sleeping due to nightmare anticipation and lack of rest, leading to diurnal dysfunction because of the excessive somnolence, fatigue, irritability, feelings of helplessness and difficulties in concentrating (Martinez et al. 2005; Langston et al. 2007).

Nightmares may be due to genetic, organic or psychological factors. Psychological stress often accompanies the occurrence of nightmares, for example typical life events that are stressful in nature can create enough significant anxiety to cause nightmares (Langston 2007).

After a severe trauma, the dominant emotions of the subject are clear, and even though the dream images do not display the actual traumatic event, past experiences and day residues are swept up by the dominant emotions of terror, fear, or vulnerability to form the picture of the dream (Hartmann 1998). It is suggested that dreams contextualize the dominant emotions of the dreamer and that dreams after trauma or stressful events can be seen as a kind of continuity, given that when these situations are dreamt, they gradually become a part of our network (Hartmann 1998). Thus, it can be assumed that dreaming has an adaptive and quasi-therapeutic function, as do nightmares, which commonly follow trauma (Agargun et al. 2003; Hartmann 1998).

The experience of nightmares after trauma suggests that people who continue to relieve their traumatic experiences by means of their dreams still have to consolidate those events into their current schemas (Horowitz 1986). The occurrence of nightmares which replicate the event immediately after trauma is usual and occurs so the new information of the traumatic experience fuses with existing information and memories, generally transforming the nightmares’ content over time, as the trauma is integrated into the person’s life experiences (Hartmann 1996). However, if the traumatic memory is not assimilated, post-traumatic nightmares will occur (Langston 2007). In other words, the person is stuck with repetitive post-traumatic nightmares, given that the situation becomes chronic, thus disabling the process of making connections with other life’s material (Hartmann 1998). Trying to find a solution, the process is activated in the same form over and over, and it is usually related to the failure of affect regulation (Varvin 2003; Varvin et al. 2012.

Traumatic memories are intrusive and rigid areas that are dominated by non-integrated free floating emotions, which disrupt the normal process of making connections, disturbing the production of ordinary memories (Hartmann 1998; Varvin et al. 2012). Therefore, these memories will mostly come back as emotional and sensory states, given that the individual is not able to represent them verbally due to the failure of processing information on a symbolic level (that may be due to the core of Post Traumatic Stress Disorder), becoming unable to integrate traumatic experiences with other life experiences (Van der Kolk 1996). The inability of deaf people to communicate their fears and unknown experiences results in the experience of several negative emotions during or after the stressful, traumatic events, ensuring traumatisation and increasing the probability of developing a trauma-related disorder (Rosenman 2002).

## Conclusion

In summary, the deaf population is more vulnerable to suffering from traumatic experiences. They display unique trauma symptoms and, in general, experience significantly more dissociative symptoms than hearing people. However, the construct of post traumatic stress disorder is manifested differently among the deaf population as reflected in the different predictors for the disorder symptoms (Schild 2007).

Some studies have found that deaf people have dreams with more negative contents, emotions and with more aggression (Pires 2009; Pires et al. 2007). However, no research about nightmares in the deaf population has been registered.

Nightmares are considered to be markers of emotional dysregulation, which contain more unarticulated and primary affects such as fear, death and aggression (Fireman et al. 2014). By sharing the dream, feelings of conflict may be expressed, which otherwise could not be communicated (Hartmann 1998; Fonagym et al. 2012; Rego 2014). However, in the deaf population this is very unlikely to happen given the lack of social support or of clinicians competent in sign language (Freyd 1994; Schild 2007).

Given the lack of information, some statements are conjectures based on the literature found relating to other conditions and their comorbidities, such as stressful events and dissociation to nightmares. Therefore, it would be of interest to study nightmares in the deaf population, given their greater contact with stressful, traumatic experiences and lack of social support. In effect, a better understanding of this issue would help to improve the practice of clinicians and therapists, since nightmares represent a conflict in the individual’s inner world that needs to be expressed and understood.
